# Startup Drift Compensation of MEMS INS Based on PSO–GRNN Network

**DOI:** 10.3390/mi16050524

**Published:** 2025-04-29

**Authors:** Songlai Han, Jingyi Xie, Jing Dong

**Affiliations:** Research Institute of Aerospace Technology, Central South University, Changsha 410083, China; songlai.han@csu.edu.cn (S.H.); 235811007@csu.edu.cn (J.X.)

**Keywords:** MEMS, startup drift compensation, PSO-GRNN

## Abstract

The startup drift phenomenon that exists in MEMS INSs increases the navigation error, prolonging the start-up time. Aiming to resolve this problem, a startup drift compensation method based on a PSO-GRNN model is proposed in this paper. We adopted a correlation analysis to determine the input parameters of the PSO-GRNN model that mainly affect startup drift. In the process of training this model, we used the PSO algorithm to optimize the spread parameter of the PSO-GRNN model. The information transmission function between particle swarms was used to find the best spread parameter by iterative optimization, the particle’s position was mapped to the GRNN model, and the GRNN model was constructed with the optimal position of the swarm as the spread parameter. This method can effectively compensate for startup drift and improve navigation accuracy. Startup drift compensation experiments were carried out at different ambient temperatures. Compared with the MEMS INS data without compensation, the standard deviation of the MEMS INS data with the proposed method decreased by more than 80.6%, and the peak-to-peak value of the MEMS INS data decreased by over 72.7%. Compared with the traditional method, the standard deviation of the MEMS INS data compensated via this method decreased by 54.5% on average, and the peak-to-peak value decreased by 42.8% on average. Meanwhile, the performance of this method was verified by navigation experiments. With the proposed method, the speed error improved by over 36.4%, and the position error improved by over 41.1%. The above experiments verified that the method of this paper significantly improved navigation performance.

## 1. Introduction

Micro-Electro-Mechanical Systems (MEMS) are a cutting-edge, 21st century technology based on micro/nano technology [1]. They can integrate micro mechanical components, sensors, actuators, and electronic circuits on a silicon substrate. With the development of MEMS manufacturing technology, MEMS inertial sensors have been widely used in military, industrial, and even civil consumer electronics fields due to their advantages of low costs, small size, and low power consumption [2].

Despite this significant technological progress, the performance of MEMS sensors is still constrained by many factors, among which startup drift is a key factor affecting their accuracy and stability [3]. Compared with the high-precision Inertial Measurement Unit (IMU), the performance of MEMS IMUs is more affected by temperature changes [4,5]. Some sensors’ outputs may cause startup drift to occur after being started at a certain temperature, which is an unexpected offset phenomenon in the output signal which occurs within a short period of time. Gyroscope output requires a long thermal stabilization process, which cannot meet the needs of fast startup [6]. In addition, since the attitude angle is calculated by integrating angular velocity, the error caused by the startup drift of the MEMS gyroscope continues to accumulate over time, and navigation accuracy decreases subsequently. Therefore, the data processing of startup drift is extremely important [7].

In today’s research on solutions to the inertial sensor startup drift problem, a common method is to establish a mathematical model to compensate for the startup drift. Commonly used mathematical models are polynomial regression models and piecewise models [8,9,10], where the coefficients of the polynomial are generally determined by the least squares method. However, since the startup drift phenomenon is affected by many factors, its complexity is often difficult to fully characterize by simple polynomial or piecewise models, resulting in a limited compensation effect.

Neural networks have the advantage of approximating the nonlinear function at any precision [11] and are widely used in the compensation of gyroscope temperature drift. Reference [12] established a long short-term memory recurrent neural network (LSTM-RNN) model. Based on this model, gyroscope drift is compensated for, and accuracy and stability are relatively ideal. However, the LSTM network requires a large amount of training data to avoid over-fitting, and there are many parameters that need to be adjusted during the training process, resulting in high complexity in the model. In Reference [13], a temperature compensation model of an IMU based on a radial basis function neural network (RBFNN) is proposed, compensating both accelerometers and gyroscopes. However, due to the use of the empirical risk minimization (ERM) principle in the model training phase, the neural network model is sometimes unable to avoid over-fitting or under-fitting problems [14]. If the appropriate grid structure cannot be found, the model’s generalization ability will be difficult to guarantee. Later, researchers proposed that an optimization algorithm can be used to find the optimal training parameters to solve this problem. References [11,15] used the gravity search algorithm (GSA) and improved particle swarm optimization (PSO) to adjust the support vector machine (SVM) algorithm to improve regression accuracy. However, For RBFNN and SVM, the strategy of processing the nonlinear problem still needs to be improved, because both RBFNN and SVM map nonlinear problems in a low-dimensional space to high-dimensional space through radial basis function or kernel techniques so that they are linearly separable in a high-dimensional space. Therefore, their performance is highly dependent on the selection of radial basis function or kernel function, and the selection problem is also more complex. They usually need to be tested and adjusted repeatedly, which increases the complexity and uncertainty of the model. References [16,17], respectively, combined a genetic algorithm and an artificial fish swarm algorithm with a neural network to improve the accuracy of model fitting. Most of these methods proposed in the above references have been used for temperature drift compensation in a steady working state, and it is not verified whether these methods are suitable for startup drift compensation in the startup phase.

This paper mainly studied the startup drift compensation algorithm of a MEMS Inertial Navigation System (INS) and proposed a new method for modeling and compensating for the startup drift of the MEMS INS. The sample size of the startup drift data processed in this paper is limited, and the startup drift data are highly nonlinear. Compared with the LSTM-RNN mentioned in Reference [12], the GRNN used in this paper only has one spread parameter that needs to be manually adjusted in order to avoid the influence of human factors as much as possible. It has high fault tolerance and robustness, and is not limited by the number of samples. Compared with the neural networks used in References [11,13,15], the GRNN used in this paper has no need to explicitly select a radial basis function or kernel function. It predicts by calculating the distance between the input and all training samples, avoiding performance degradation caused by improper selection of kernel functions. The prediction process of the GRNN can be understood as a non-parametric regression analysis that uses the information from training samples to estimate the conditional mean of the target variable, thereby enabling the flexible modeling of nonlinear relationships. The innovations of this paper are as follows: (1) A startup drift compensation method of MEMS INS based on PSO-GRNN was proposed, which improved the network training speed and fitting accuracy. (2) It was demonstrated that temperature, temperature change rate, temperature square term, and the cross term of temperature and temperature change rate significantly affected on the startup drift. The above physical quantities were used as model inputs to complete the modeling process.

The structure of this paper is as follows: In Section 2, the causes and complexities of the startup drift phenomenon are analyzed, followed by an investigation of the impact of startup drift on navigation accuracy through simulation. In Section 3, a MEMS INS startup drift compensation method based on a generalized regression neural network (GRNN) is proposed. The GRNN is used to model the startup drift, and we use the particle swarm optimization (PSO) algorithm to find the optimal spread parameter. In Section 4, based on the model obtained in the previous section, data compensation experiments and navigation experiments are carried out to verify the effectiveness of the compensation model. Section 5 concludes the paper.

## 2. Analysis of Startup Drift Characteristics of MEMS INS

**Figure 1:**
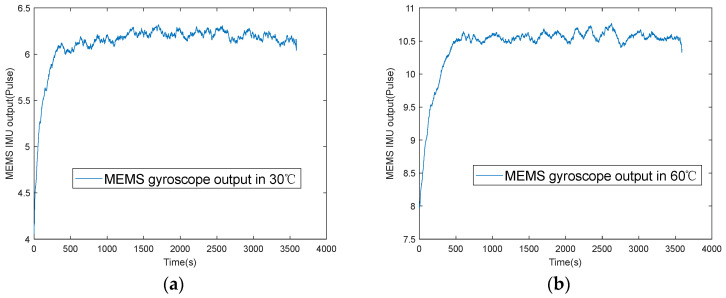
MEMS gyroscope cold start output T different temperature points: (**a**) 30 °C; (**b**) 60 °C.

The startup drift of MEMS INS is mainly manifested by the obvious deviation or drift of the sensor’s output in a short time after the start. Figure 1 shows the output data of a certain axis during the cold start of a MEMS gyroscope at 30 °C and 60 °C, respectively.

From these figures, we can observe that the output of this MEMS gyroscope goes through two stages. The first stage is the “startup stage”. The gyroscope output is highly unstable in the startup stage, and startup drift occurs. This instability is because the material of MEMS sensors is silicon, the silicon material is very sensitive to temperature, and its performance changes significantly with the temperature of the external thermal environment [18]. After powering on, a large amount of heat is generated in the internal unit, resulting in a dramatic change in the thermal environment, which causes drift error in the gyroscope output. This type of drift error is called startup drift.

The gyroscope output enters a steady working stage at about 600 seconds. During this stage, the gyroscope output exhibits a drift phenomenon caused by temperature changes, referred to as temperature drift in the steady state (temperature drift for short). As shown in Figure 1a,b, we find that temperature drift varies at different start-up temperature points [19].

The temperature excitation of the startup drift is different from that of the temperature drift. The temperature changes observed during the startup stage primarily result from the self-heating of the internal components of the sensors. In contrast, the characteristics of these internal components in the steady working stage are mainly influenced by the external ambient temperature [20]. Compared with the temperature characteristics of the temperature drift in the steady stage, the temperature characteristics of the startup drift are more complex, exhibit weaker regularity, demonstrate poor repeatability, and are more challenging to model accurately. When startup drift occurs in the sensors’ output, the range and rate of temperature change can be substantial, resulting in significant alterations to the physical properties of the sensors (such as material thermal conductivity, thermal expansion and contraction, etc.), which in turn affects the drift characteristics of the sensors. In contrast, after the output reaches a steady state, the temperature changes become relatively small and gentle, and the change in the physical characteristics is relatively steady, so the temperature characteristics of the temperature drift are simpler to analyze. And the startup drift phenomenon varies significantly at different temperature points, making it challenging to develop a compensation model that applies universally across all temperatures. In general, the temperature excitation of the startup drift and the temperature drift in the steady state are very different, and the models at different temperature points are also different. Therefore, it is more difficult to realize the startup drift compensation than the temperature drift.

**Figure 2:**
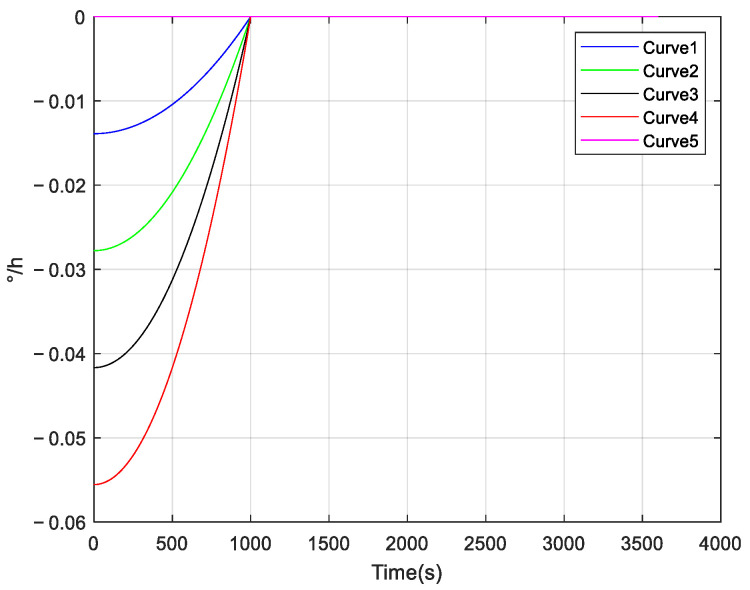
Start-up drift added in the simulation process.

When we use INS to navigate, in order to shorten the preparation time of alignment and navigation, higher demands are placed on the startup performance of the inertial sensors. However, the phenomenon of startup drift can introduce errors in both alignment and navigation, significantly affecting accuracy and real-time performance. The following simulation is performed to show the impact of startup drift on navigation accuracy, and further emphasizes the importance of compensating for startup drift. The simulation process is as follows: the INS is in a stationary state, and five groups of startup drifts are added as shown in Figure 2. It is assumed that the duration of each startup drift is 1000 seconds, the initial velocity is 0 m/s, and the initial roll angle, pitch angle, and heading angle are all 0°. The simulation duration in the pure inertial navigation mode is one hour. Based on the calculated navigation results, we have investigated the impact of startup drift on navigation accuracy. The startup drifts we added during the simulation process are shown in Figure 2.

**Figure 3:**
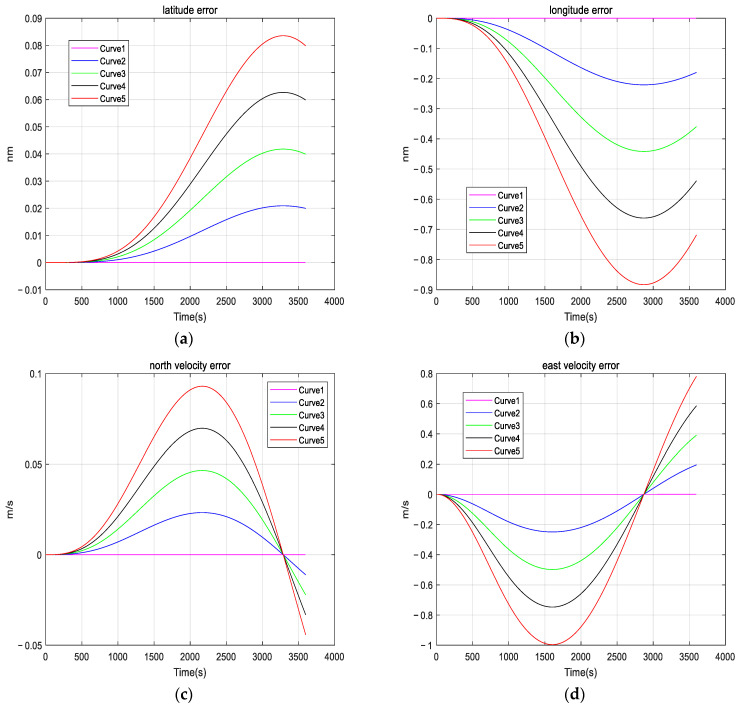

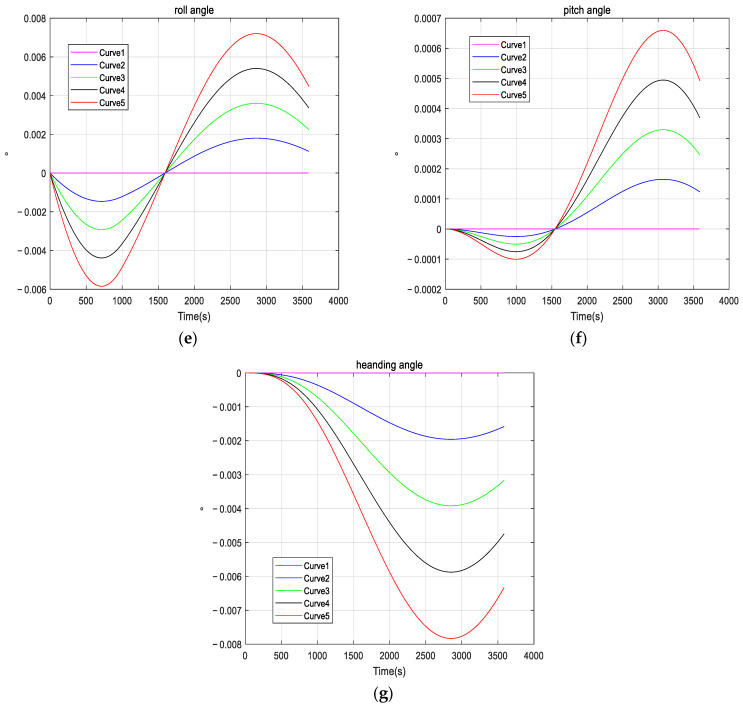
Navigation error under different startup drifts: (**a**) latitude error under different startup drifts; (**b**) longitude error under different startup drifts; (**c**) north velocity error under different startup drifts; (**d**) east velocity error under different startup drift; (**e**) roll angle error under different startup drift; (**f**) pitch angle error under different startup drift; and (**g**) heading angle error under different startup drift.

Figure 3 shows the navigation results; we can observe that startup drift can lead to navigation errors. Consequently, compensating for startup drift is essential.

## 3. Startup Drift Compensation Method of MEMS INS Based on PSO-GRNN

In the previous section, through simulation calculations, we found that the startup drift will cause navigation errors during the inertial navigation process, which will affect the normal use of the device, so the startup drift cannot be ignored [17]. In this paper, the GRNN is used to model the startup drift of MEMS INSs. We use particle swarm optimization (PSO) to optimize the spread parameter of the GRNN so that the trained model has better fitting and generalization capabilities for MEMS INSs startup drift.

### 3.1. Generalized Regression Neural Network Structure

The Generalized Regression Neural Network (GRNN) is a probabilistic learning algorithm characterized by a single channel and parallel memory structure. The GRNN was originally proposed by Specht [21], as an improvement on the Radial Basis Function network (RBF). GRNN can self-train in a short time, and it is a one-way propagation neural network without backward propagation or iteration. The performance of the GRNN can be adjusted only by setting the spread parameter, thereby minimizing the influence of human factors. It overcomes the shortcomings of traditional learning algorithms, such as complex structure design and parameter sensitivity, and has the advantages of fast convergence speed, high prediction accuracy, not easily falling into a local minimum, high fault tolerance, and high robustness. It is not limited by the number of samples; even with a limited dataset, the effect of regression predictions remains strong [22]. Based on these advantages, the GRNN is widely used in function approximation and regression prediction in various fields.

**Figure 4:**
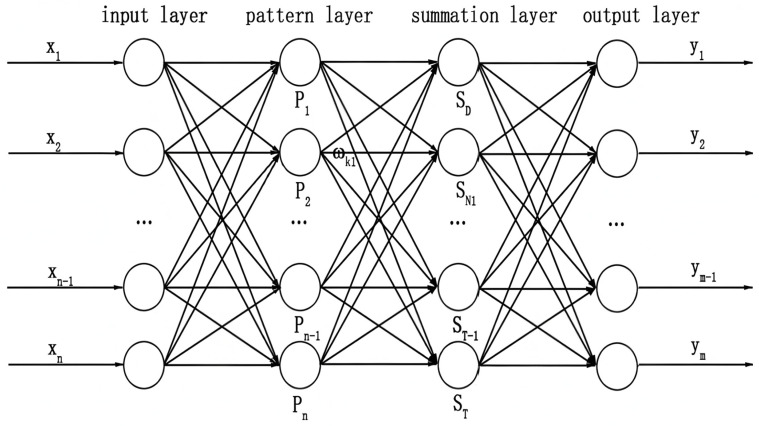
GRNN neural network structure diagram [25].

The GRNN model is a highly parallel radial basis network [23,24], as shown in Figure 4, which consists of four layers: a data input layer, a pattern layer, a summation layer, and an output layer.

The input layer receives the input data and passes it to the next layer.

The pattern layer uses the Gaussian function to process the input data [26], as shown in Equation (1). $x_{i}$ is the training sample, $x_{j}$ is the learning sample corresponding to the *i*th neuron, and σ is the spread parameter, which is the only parameter that needs to be artificially set in the whole network. Whether it is reasonably set or not has a direct impact on the prediction performance of the model. It being too large or too small will lead to under-fitting and over-fitting of the network, respectively. The determination of the spread parameter is essentially an optimization problem, that is, a problem of finding an optimal value so that the mean square error between the predicted output value and the actual value is the smallest [27]. The most commonly used methods for this optimization are generally the holdout method, described by Specht [28], and the fruit fly optimization algorithm, the particle swarm optimization algorithm, and so on, which can be used for parameter optimization to ensure the generalization capability and accuracy of the model [29].

(1) $$g_{i}=\exp(-\frac{\left\|x_{i}-x_{j}\right\|}{{2\sigma }^{2}})$$

The summation layer uses two types of neurons for summation [28]. $S_{D}$ is the arithmetic summation of all pattern layer outputs, as shown in Equation (2), and $S_{\mathrm{Nj}}$ is the weighted summation of all pattern layer outputs, as shown in Equation (3), where $y_{\mathrm{ij}}$ is the *j*th element in the *i*th output sample $Y_{i}$.

(2) $$S_{D}=\sum_{i=1}^{n}g_{i}$$

(3) $$S_{Nj}=\sum_{i=1}^{n}y_{ij}g_{i}$$

The output value of the summation layer is converted and output in the output layer. According to the calculation results of the summation layer, the output is calculated:

(4) $$O_{j}=\frac{S_{Nj}}{S_{D}}$$

Compared to the neural network methods mentioned in the Introduction, the advantages of GRNN in terms of computational complexity and real-time performance are as follows:

① The GRNN is a forward operation, which only needs to calculate the Euclidean distance between the test point and the training sample and the Gaussian weighted average when predicting. It does not involve recursive operation, a single running time is short, no time series dependence, and each prediction is processed independently. In contrast, the LSTM-RNN needs to be calculated in time step order [30], which cannot be parallelized and has poor real-time performance. The computational complexity of the LSTM-RNN mainly comes from the matrix operation of temporal expansion and the gating unit: each time step needs to calculate the activation of the forgetting gate, input gate, and output gate; the calculation of the current step depends on the hidden state of the previous step [31]; therefore, the LSTM-RNN is more suitable for modeling time series problems. In this paper, the time dependence of startup drift is very small, and the historical value has little impact on the current value. In contrast, the computational complexity of the GRNN is lower than that of the LSTM-RNN, because it directly uses the analytical solution of the training sample (Parzen window estimation) without back propagation.

② Because the SVM needs to solve a quadratic programming problem [32] and select the kernel function, while the GRNN only needs to find the optimal spread parameter, the computational complexity of the SVM during the training process is higher than that of the GRNN. In terms of real-time performance, the prediction speed usually depends on the ratio of the number of support vectors to the number of samples. If the number of support vectors is much lower than the number of samples (such as the linear SVM), the SVM prediction will be more efficient [33]; if the number of support vectors is close to the number of samples (complex nonlinear), the prediction efficiency of both methods will be equivalent, but, because the GRNN can be operated in parallel, the GRNN is faster overall; the data to be fitted in this paper is highly nonlinear. The number of support vectors in the SVM may reach 80% of the number of samples, so the parallel computing of the GRNN may be faster.

③ Compared with the RBF, the GRNN has more advantages in terms of computational complexity during the training phase because the GRNN needs to adjust only one parameter, while the RBF needs to adjust the clustering center, weight, and the width of the Gaussian kernel [34]. In terms of real-time performance, the GRNN is slightly inferior to the RBF, because, in the prediction stage, the calculation amount for the RBF depends on the number of hidden layer nodes, while the GRNN relies on the entire training data. However, both of them can complete the operation on an industrial personal computer, and the runtime is close. From the perspective of real-time performance, although the advantages of the GRNN are not obvious compared with the RBF, it has more advantages in nonlinear mapping ability and learning speed.

### 3.2. Parameter Optimization Based on PSO

It was mentioned in the previous section that the value of the spread parameter directly influences the accuracy and generalization capability of the model. Therefore, we adopt the particle swarm optimization algorithm to optimize the spread parameter and improve the fitting ability of our model.

Particle swarm optimization (PSO) is an evolutionary computing technology, which was proposed by Dr. Eberhart and Dr. Kennedy in 1995, originating from the behavioral study of bird flocks’ predation patterns [35]. PSO simulates the predation behavior of bird flocks. The particles adjust their positions and velocities, based on their own experience and group experience by transmitting information to each other and gradually approaching the optimal solution. The updating formulas for position and velocity are shown in Equations (5) and (6). The evaluation of the optimal solution is achieved by calculating the fitness function, which, in this paper, is chosen as the mean square error (MSE). This is the mean square error between the predicted values of the samples and the actual values, as shown in Equation (7), a smaller MSE indicates a better model fit.

(5) $$V_{i}^{k+1}=w*V_{i}^{k}+c_{1}r_{1}(P_{best}^{k}-P_{i}^{k})+c_{2}r_{2}(G_{best}^{k}-P_{i}^{k})$$

(6) $$x_{i}^{k+1}=x_{i}^{k}+v_{i}^{k+1}$$

(7) $$MSE=\frac{1}{n}\sum_{i=1}^{n}{\left(y_{p}-y_{i}\right)}^{2}$$

where the subscript i denotes the i th particle and the superscript k denotes the k th iteration, r_1_ and r_2_ are both random numbers within the range [0, 1], c_1_ and c_2_ are learning factors, w is inertia weight, x represents position, V represents velocity, P_best_ represents the individual optimal position, G_best_ represents the optimal position of the group, y_p_ is the value predicted by the neural network, and y_i_ is the actual value.

**Figure 5:**
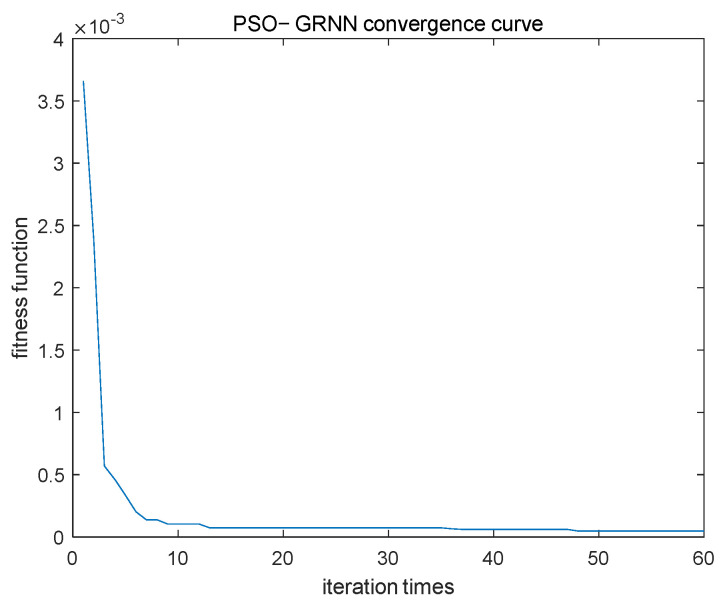
PSO-GRNN convergence curve.

In this method, the optimization results of the PSO algorithm mainly depend on the number of iterations. As shown in Figure 5, when the number of iterations is less than 10, the fitness function value decreases rapidly, and once the number of iterations exceeds 10, the fitness function value converges slowly and tends to become stable. In order to make the PSO results converge as much as possible, the number of iterations is set to 30 in this paper. In addition, after many experiments, it was found that when the number of iterations reaches 30, the optimization results are not sensitive to the selection of other parameters, and the difference in fitness function values is very small, and they can all meet the accuracy requirements. The settings for the other parameters in this paper are as follows:

The number of particles is set to 30, the number of iterations is set to 30, the inertia weight w is set to 0.8, and the learning factors c1 and c2 are both set to 1.5.

After completing this algorithm, the particle’s position with the optimal fitness (G_best_) is output, completing the process of optimization. G_best,_ in this paper, is the spread parameter that makes the GRNN model fit best.

### 3.3. Startup Drift Compensation Method of MEMS INS Based on PSO-GRNN

**Figure 6:**
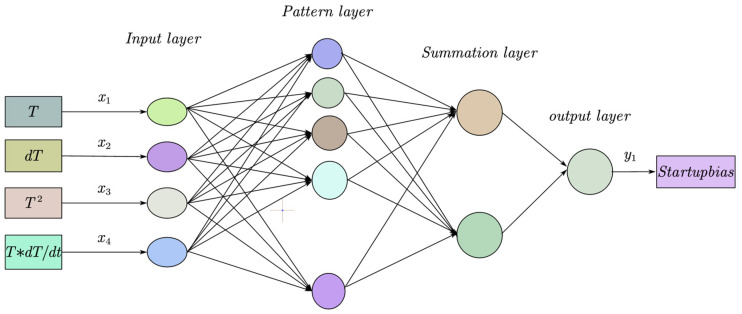
Diagram of the PSO-GRNN-based startup drift compensation model.

Through literature research and experimental observation, it has been found that. during the startup process of MEMS INSs, the temperature terms that affect the startup drift usually involve temperature, the temperature square term, the change rate of temperature, the spatial gradient of temperature, etc. [36]. Some studies also incorporate time as a model input parameter [37,38]. However, if all these parameters are considered as model inputs, the model will become very complex, and the generalization capability will be reduced. Correlation analysis can be employed to determine the model input parameters that have a major impact on the startup drift of the inertial sensors and improve the generalization of the model [39]. According to the calculation results of the correlation coefficients, this paper considered that the correlation coefficients of the temperature term, the quadratic term of temperature, and the rate of temperature were large. In addition, Reference [40] considers that incorporating the coupling term of the temperature and the rate of temperature as the model input will have a certain promotional effect on improving the model’s compensation capability. In summary, the inputs of this compensation model established in our paper include the temperature term, the temperature square term, the rate of temperature, and the coupling term of the temperature and the temperature change rate, as shown in Figure 6.

After determining the input parameters of the model, we need to preprocess the original data, because the original output of inertial sensors contains a significant amount of noise, which can directly impact the accuracy of the model. Therefore, we chose to eliminate the noise by 100 s smoothing. In addition, the input and output parameters of the model need to be normalized to avoid the adverse effect of singular sample data on the accuracy of the model. Then, in order to better compare the fitting results, the output predicted by the model was reverse-normalized after the modeling was completed. In this paper, we employed the maximum and minimum method for normalization. The formula is as follows:

(8) $$x=\frac{x_{i}-x_{\min}}{x_{\max}-x_{\min}}$$

The model directly outputs the predicted startup drift value based on the temperature-related term. Usually, the output enters the stabilizing stage after a period of startup, and the output data are roughly steady and no longer contain the startup drift term. Therefore, the average output of the inertial sensors in the steady state is subtracted from the original output data, and the startup drift term is obtained.

We use the GRNN to fit the startup drift of MEMS INS. The fitted startup drift is as follows:

(9) $$A=f(T,T^{2},dT/dt,T*dT/dt)$$

A is the startup drift to be fitted, and f is the trained model.

**Figure 7:**
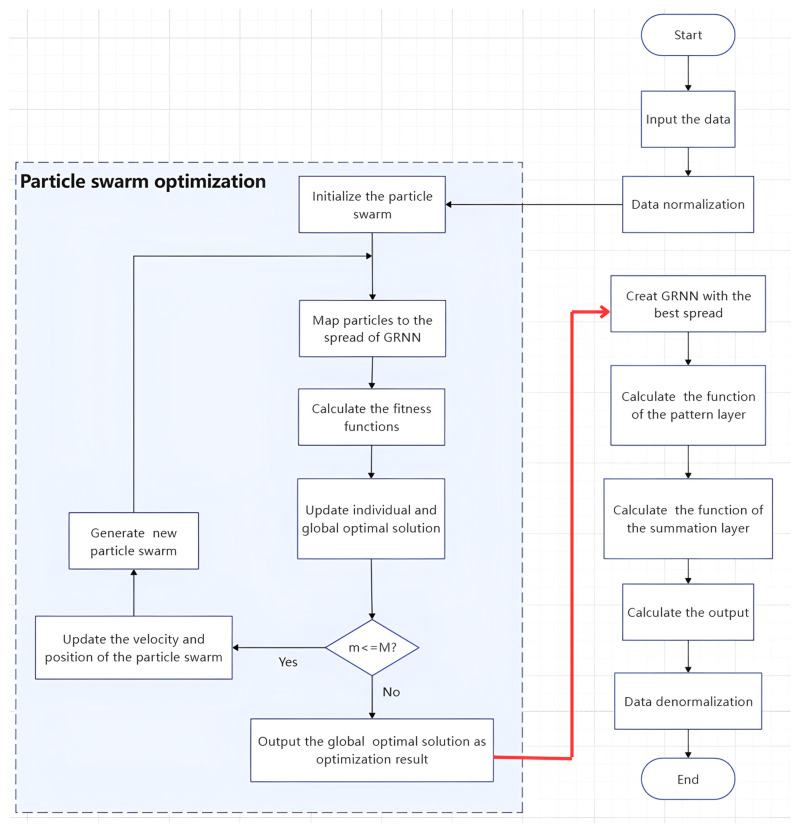
Flow chart of this paper’s methodology.

The flow chart for the use of the PSO-GRNN to fit the startup drift of MEMS INS is presented in Figure 7. The process of obtaining this model is as follows: ① Data preprocessing; ② after determining the input and output parameters of the network, we calculated the spread parameter; according to the collected training set, the GRNN model is trained. In the process of training the model, we used the PSO algorithm described in Section 3.2 to find the optimal spread parameter by minimizing the fitness function, as in the left half of Figure 7. ③ We substituted the spread parameter value into the GRNN model, and the trained model was obtained. In practical applications, we used the trained model to calculate the predicted startup drift and compensate for it by feeding the collected temperature and its associated terms into the model’s input layer.

The specific process of the PSO of the spread parameter of the GRNN in Step 2 is as follows:

(a) We select the mean square error (MSE) between the actual values and the predicted values as the PSO fitness function. The purpose of each PSO iteration in updating the spread parameter in the GRNN model is to find the minimum MSE.
(b) Initialize the velocities and positions of the particles, and define the optimization parameters of the optimization algorithm; the particle’s position is mapped to the GRNN model, and the GRNN model is constructed with G_best_ as the spread parameter; if the fitness is better than the previous optimal fitness, P_best_ and G_best_ are updated.
(c) Determine whether the maximum number of iterations has been reached. If it has been reached, the optimal G_best_ found by the particle swarm is the optimal spread parameter. Save the optimal GRNN model and predict the test set; if not, return (b).

Among them, the optimization parameters of the optimization algorithm in (b) are set as follows:

The number of particles is set to 30, the number of iterations is set to 30, the inertia weight w is set to 0.8, and the learning factors c1 and c2 are both set to 1.5.

## 4. Experimental Verification and Analysis

In order to verify whether the method presented in this paper can effectively compensate for the startup drift, we carried out the data compensation experiment and the navigation experiment for analysis and verification. In the data compensation experiment of MEMS INS, we collected the raw data at various temperature points, then applied the proposed compensation model to fit the startup drift. The fitted startup drift was subtracted from the raw data to obtain the compensated data. Two sets of data without and with compensation were compared to verify whether the startup drift had been effectively compensated. In the navigation experiment, we used the raw data from the inertial sensors and the compensated data to perform the inertial navigation, and analyzed the navigation error to verify whether the compensation of the startup drift enhanced the navigation accuracy.

### 4.1. Data Compensation Experiment of Inertial Sensors

In the data compensation experiment, we placed the MEMS INS in the vibration isolation foundation of a temperature chamber, and the INS remained completely stationary in the entire test process. The following six start-up temperature points were set in the experiment: 10 °C, 20 °C, 30 °C, 40 °C, 50 °C, and 60 °C. At each start-up temperature point, the MEMS INS was insulated for two hours without startup, and then powered on to collect the raw data for one hour; the raw data frequency was 1000 Hz. After that, the raw data were processed by the method mentioned in Section 3.3. The performance indicators of MEMS INS are presented in Table 1.

**Figure 8:**
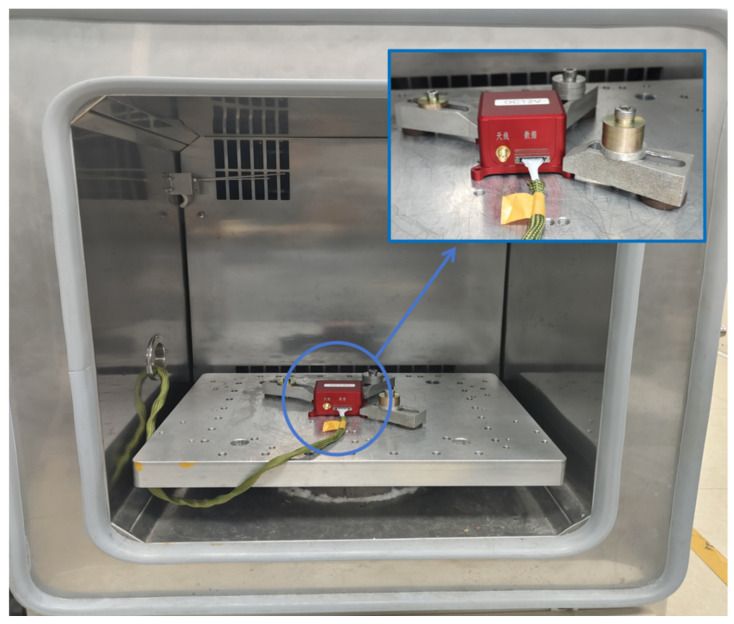
Data acquisition process diagram.

The data collection process is shown in Figure 8:

In this paper, the performance of the proposed compensation algorithm is evaluated by selecting a commonly used multiple regression compensation model as the control group, and comparing the compensation effect of the proposed method with that of the multiple regression model. The multiple regression model first determines the function type of the empirical formula, and then uses the least squares method to determine the normal equations. By solving these normal equations, the coefficients of the regression terms are obtained. The regression model of inertial sensors’ drift that is commonly used in various applications is:

(10) $$StartupBias=a_{0}T+a_{1}T^{2}+a_{2}dT/dt$$

where *a*_0_, *a*_1_, and *a*_2_ are the coefficients of different temperature terms. To ensure fitting accuracy, the multiple regression compensation model is fitted separately for each temperature point. Next, we compare the compensation effects of the PSO-GRNN model and the traditional model: two compensation models are used to compensate for startup drift separately at different temperature points, and the compensation effects of the two methods are presented.

**Figure 9:**
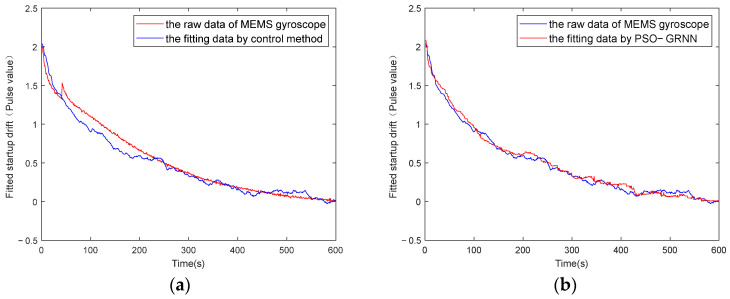
Gyroscope startup drift and its fitting data when starting at 50 °C: (**a**) fitting with the control group; (**b**) fitting with the proposed method.

**Figure 10:**
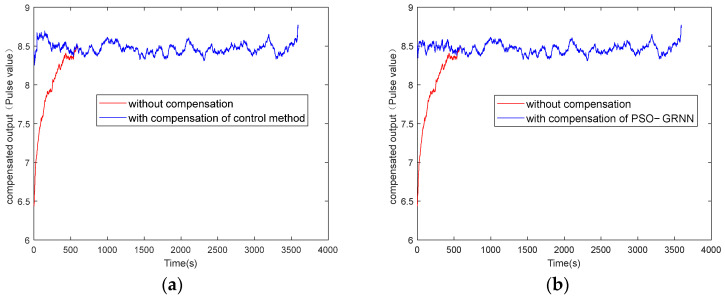
Gyroscope startup drift data with and without compensation at 50 °C: (**a**) compensation with the control group; (**b**) compensation with the proposed method.

**Figure 11:**
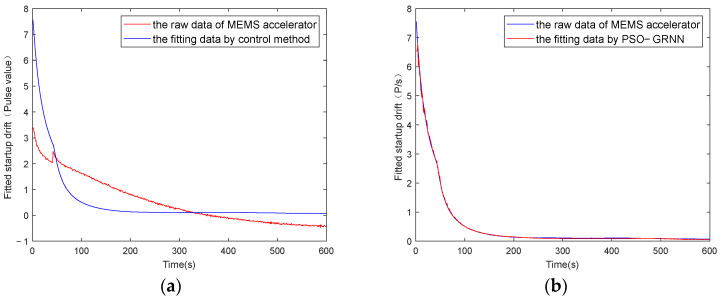
Accelerometer startup drift and its fitting data when starting at 50 °C: (**a**) fitting with the control group; (**b**) fitting with the proposed method.

**Figure 12:**
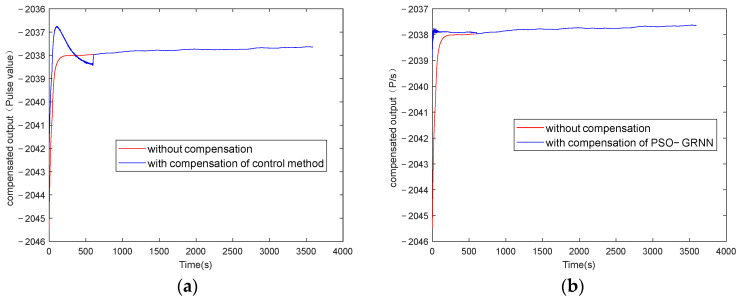
The accelerometer startup drift data with and without compensation at 50 °C: (**a**) compensation with the control group; (**b**) compensation with the proposed method.

The y-axes of Figure 9, Figure 10, Figure 11 and Figure 12 represent the pulse values output by the inertial sensors, where the nominal scale of the gyroscope is 1.08, and the nominal scale of the accelerometer is 2000. Therefore, the gyroscope output with a pulse value of 8.5 in Figure 10 is approximately equivalent to 7.87°/h, and the accelerometer output with a pulse value of 2038 in Figure 12 is approximately equivalent to 9.8 m/s^2^ (1 g).

Figure 9 and Figure 11 show the absolute values of startup drift and the corresponding fitted data during the cold start of a MEMS gyroscope and an accelerometer at an ambient temperature of 50 °C, respectively. Figure 10 and Figure 12 show the comparative results of compensating for startup drift during the cold start of a MEMS gyroscope and an accelerometer at an ambient temperature of 50 °C, respectively. The acquisition process for startup drift shown in Figure 9 and Figure 11 is as follows:

Taking the gyroscope as an example, the red curve in Figure 10 represents the raw data of the gyroscope without compensation. It indicates that the gyroscope’s output is highly unstable during the initial startup phase, with a significant absolute value of the temperature change rate observed during this period, resulting in considerable startup drift. The gyroscope output data fluctuate slightly around a certain mean value after 600 seconds. It is considered that the startup drift approaches zero, representing the ideal data without startup drift. Therefore, the startup drift can be obtained by subtracting the mean value of the output data in the steady state from the output data of the start-up state, then we use the GRNN model proposed in this paper and the traditional multiple regression model to fit the startup drift, respectively. Finally, the ideal data without startup drift is obtained by subtracting the fitted startup drift from the original output of the gyroscope. As shown in Figure 11, the model proposed in this paper can more accurately fit the startup drift. From Figure 12, it can further be found that the fitting effect of our method is more accurate, and the compensation effect is better. After applying this method, the startup drift is nearly eliminated, and the data stability of the compensated output is significantly improved.

To thoroughly describe the compensation effect of our method, and compare it with the multiple regression model, Table 2 and Table 3, respectively, summarize the standard deviation (index-1) and peak-to-peak (index-2) values of the uncompensated and compensated data of inertial sensors. In this paper, the calculation of standard deviation and peak-to-peak values was to accumulate the data with sampling frequency of 1000 Hz to generate sampling data with interval of 1 s, and calculate the standard deviation of these 600 data points within 600 seconds. We actually calculate the dispersion of these 600 data points. With our method, the stability of the inertial sensors’ output during the startup process is significantly enhanced.

From Table 2, it can be seen that index-1 of the compensated gyroscope output was reduced by at least 80.6%, and index-2 was reduced by more than 73.7%. Compared with the traditional multiple regression model established for each temperature point, index-1 of the gyroscope output with the GRNN compensation model of this paper was basically smaller than the output with the multiple regression compensation model, which was improved by 17.0% on average, and index-2 was improved by 9.5% on average. It can be seen from Table 3 that with compensation, index-1 of the accelerometer output was reduced by more than 92.3%, and index-2 was reduced by over 72.7%. Compared with the traditional method, index-1 of the accelerometer output with compensation in this paper was reduced by more than 89.3%, and index-2 was reduced by more than 64.12%. Based on the results of Table 2 and Table 3, the startup drift of the MEMS INS was greatly reduced, and the stability of the sensors’ output was also improved; the model of this paper was obviously more accurate than the traditional multiple regression model.

According to the data compensation experiment of the MEMS INS, the PSO-GRNN compensation algorithm can effectively compensate for the startup drift during the cold start process, and enhance the output stability, with a significant compensation effect.

### 4.2. Navigation Experiment

In order to verify whether this method is effective, we conducted navigation experiments; the collected raw data were compensated by the multiple regression model described in Section 4.1 (i.e., method 1 in the graph) and the method in this paper (method 2), then obtained two groups of compensated data. The experiment process of the navigation experiment is as follows: The data collected in Section 4.1 was used for navigation calculation. The navigation calculation process entailed the following: 1 min of alignment, and inertial navigation after the alignment. The total time was 10 min. We calculated the navigation results of two sets of data, and their navigation errors were compared to verify whether the compensation method was effective or not.

**Figure 13:**
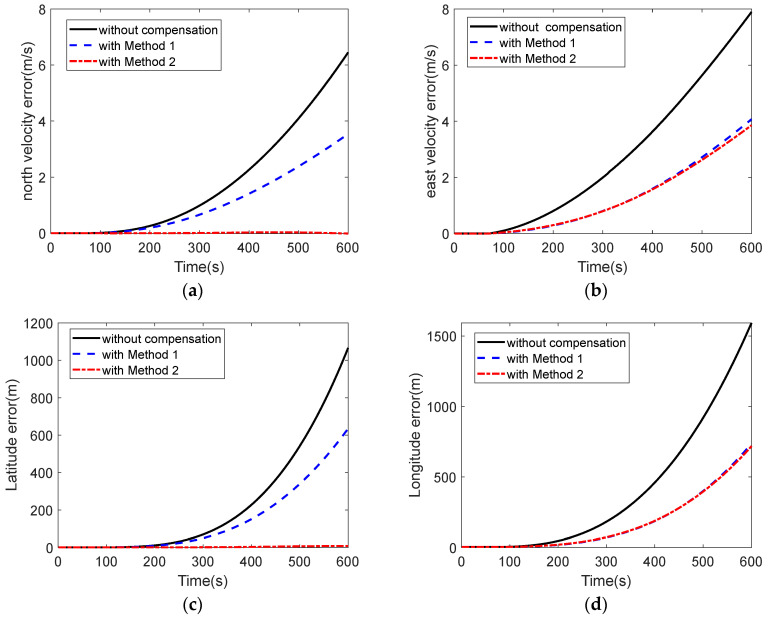

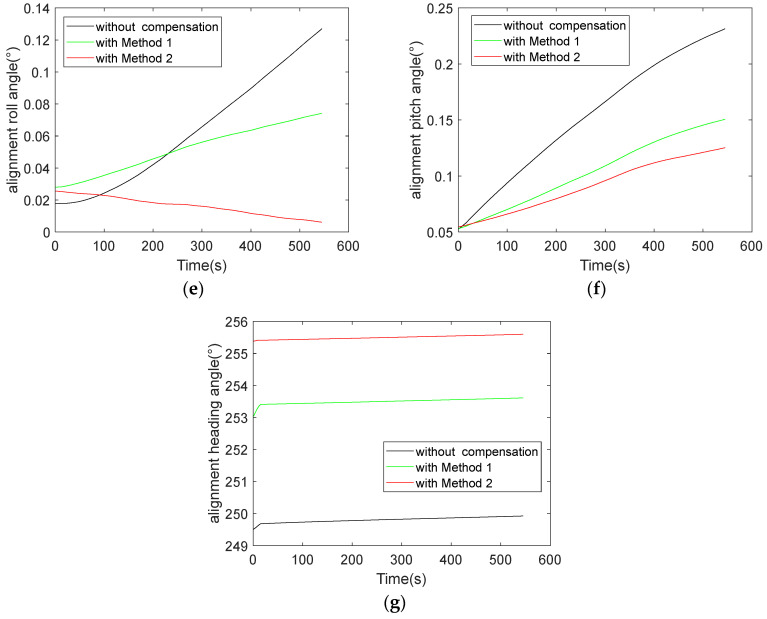
Navigation results at 20 °C: (**a**) north velocity error of the data with and without compensation; (**b**) east velocity error of the data with and without compensation; (**c**) latitude error of the data with and without compensation; (**d**) longitude error of the data with and without compensation; (**e**) alignment roll angle of the data with and without compensation; (**f**) alignment pitch angle of the data with and without compensation; (**g**) and alignment heading angle of the data with and without compensation.

Figure 13 shows the errors in the inertial navigation process when the data without compensation, compensated by multiple regression model and compensated by PSO-GRNN model, were computed, respectively, during a cold start in a 20 °C environment. Figure 13a shows the north velocity error, and Figure 13b shows the east velocity error. The maximum velocity error of the data without compensation was 7.8942 m/s, and the maximum north velocity error was 6.4523 m/s. The maximum east velocity error with the compensation of method 1 (multiple regression model) was 4.0719 m/s, and the maximum north velocity error was 3.5430 m/s. The maximum east velocity error was 3.8596 m/s, and the maximum north velocity error was −0.0115 m/s with the compensation of method 2 (this paper’s method). With the PSO-GRNN model compensation, the velocity error was greatly suppressed. Figure 13c,d show the latitude error and longitude error in the process of inertial navigation calculation, respectively. The longitude error of the data without compensation was 0.8598 nm and the latitude error was 0.5760 nm. The longitude error was 0.3978 nm and the latitude error was 0.3432 nm with the compensation of method 1. With the compensation of method 2, the longitude error was 0.3877 nm and the latitude error was 0.0044 nm. With the compensation, the positioning accuracy of MEMS INS was significantly improved. Figure 13e–g show the alignment roll angle, pitch angle, and heading angle; due to the lack of real attitude angle reference during the experiment, we can only judge the alignment accuracy by observing the navigation results of velocity and position. Compared with method 1, the method of this paper is more effective in reducing navigation errors.

Table 4 shows the navigation errors derived from the inertial navigation calculation of the uncompensated and compensated data with experiments conducted at six temperature points ranging from 10 °C to 60 °C. We can see from Table 4 that starting at various temperature points, compared with the uncompensated results, the compensated data undergoes navigation calculation resulted in the lower velocity error and position error, the velocity error of the compensated method was improved by more than 36.4%, and the position error was improved by more than 41.1%.

**Figure 14:**
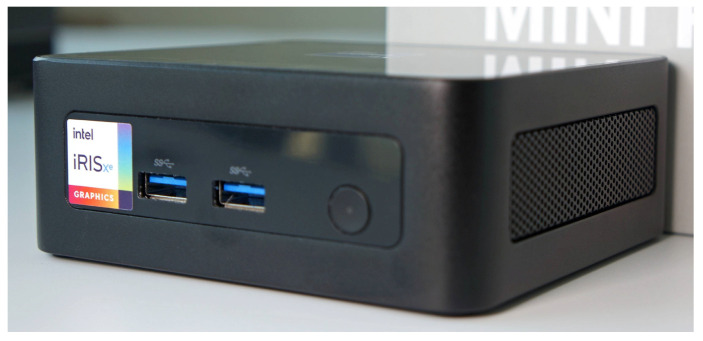
IPC used in this paper.

The proposed method can be implemented on the industrial personal computer (IPC). The IPC selected in this paper is shown in Figure 14. The processor used in the industrial computer is a 13th-generation Intel Core i7-1360P. The algorithm in this paper can complete an operation within 8.9 ms, and the control method completes the same task within 6.3 ms. Although the time used by this method is slightly longer than that of the control method, both of them can be completed on the IPC. In terms of fitting accuracy and enhancing navigation precision, the method in this paper is obviously better than the control method, so the startup drift compensation method proposed in this paper proves to be more practical.

Although the proposed method effectively compensates for startup drift and enhances navigation accuracy, it has limitations, particularly regarding real-time application. The PSO-GRNN method in this paper can finish its running on the IPC, and can be completed within 10 ms. However, due to its reliance on a neural network for modeling, it involves a large number of multiplication and division operations; this results in a higher computational complexity compared to polynomial fitting, rendering it currently unsuitable for real-time operating systems like those in single-chip microcomputers. Therefore, real-time implementation represents a critical area for further research. In the future, methods such as upgrading the chip performance of embedded systems can be used to facilitate its application in real-time systems.

## 5. Conclusions

We analyzed the phenomenon of MEMS startup drift and proposed a startup drift compensation method based on a generalized regression neural network, which took temperature, temperature square term, temperature change rate, the coupling term of temperature, and the temperature change rate as network inputs. This paper described, in detail, the modeling steps and the compensation process of this method. In order to verify the effectiveness of this method on compensating for the startup drift, the original data compensation experiment and navigation experiment at various temperature points were both carried out. The standard deviation of the compensated gyroscope output was reduced by at least 80.6%, and the peak-to-peak value by at least 73.7%. The standard deviation of the accelerometer output was reduced by at least 92.3%, and the peak-to-peak value by at least 72.7%. Compared with the traditional method, the standard deviation and peak-to-peak values of the inertial sensors’ output based on the generalized regression neural network model were enhanced significantly. Compared with the navigation results of uncompensated data, the velocity error of the compensated method was improved by more than 36.4%, and the position error was improved by more than 41.1%, which verified that the proposed method can effectively compensate for the startup drift and shorten the start-up time.

## Figures and Tables

**Table 1:** MEMS INS performance indicators.

Inertial Sensor	Parameter	Value
Gyroscope	Bias Repeatability (°/h)	0.5
	Scale Factor Repeatability(10^−6^)	<50
	Scale Factor Nonlinearity (10^−6^)	<100
Accelerometer	Bias Repeatability (mg)	<3.5
	Noise Density (μg/sqrt(Hz))	25
	Scale Factor Nonlinearity (10^−6^)	<1000

**Table 2:** The standard deviation and peak-to-peak value of MEMS gyroscope output with and without compensation at different startup temperature points.

Temperature	Standard Deviation			Peak–Peak		
	Without Compensation	Regression Compensation	GRNN Compensation	Without Compensation	Regression Compensation	GRNN Compensation
10 °C	0.4232	0.0561	0.0441	1.6005	0.2645	0.3114
20 °C	0.4560	0.0674	0.0655	1.9076	0.4388	0.3274
30 °C	0.4636	0.1007	0.0897	2.0251	0.6186	0.5315
40 °C	0.4631	0.0788	0.0573	1.9837	0.4364	0.3071
50 °C	0.4533	0.0771	0.0519	2.0656	0.4792	0.3331
60 °C	0.6975	0.0747	0.0695	2.6539	0.3188	0.3979

**Table 3:** The standard deviation and peak-to-peak value of MEMS accelerometer output with and without compensation at different startup temperature points.

Temperature	Standard Deviation			Peak–Peak		
	Without Compensation	Regression Compensation	GRNN Compensation	Without Compensation	Regression Compensation	GRNN Compensation
10 °C	1.5275	0.5627	0.0494	9.1468	3.9902	1.2466
20 °C	1.4005	0.8847	0.0557	8.6076	5.8035	0.9565
30 °C	1.3303	0.8549	0.0438	8.1978	4.9106	0.7428
40 °C	1.2409	0.8247	0.0732	7.7829	5.4523	1.2536
50 °C	1.1831	0.8030	0.0667	7.4888	5.3297	1.1677
60 °C	1.0665	0.7701	0.0818	6.9840	5.3081	1.9044

**Table 4:** Inertial navigation results at different temperature starting points.

Ambient Temperature	Without Compensation		With Compensation	
	Speed Error (m/s)	Position Error (nm)	Speed Error (m/s)	Position Error (nm)
10 °C	8.9452	0.8998	1.4085	0.1350
20 °C	10.1956	1.0349	3.8596	0.3871
30 °C	4.2967	0.3779	0.9806	0.0180
40 °C	8.1953	0.8719	4.3824	0.4522
50 °C	8.2630	0.8621	4.3616	0.4240
60 °C	2.6163	0.3177	1.6629	0.1869

## Data Availability

The dataset is available on request from the authors.
